# Health Risk Assessment of Dietary Cadmium Intake: Do Current Guidelines Indicate How Much is Safe?

**DOI:** 10.1289/EHP108

**Published:** 2017-03-01

**Authors:** Soisungwan Satarug, David A. Vesey, Glenda C. Gobe

**Affiliations:** 1 Centre for Kidney Disease Research, Translational Research Institute, University of Queensland School of Medicine, Woolloongabba, Brisbane, Queensland, Australia; 2 Department of Molecular Biology and Applied Physiology, Tohoku University School of Medicine, Sendai, Japan

## Abstract

**Background:**

Cadmium (Cd), a food-chain contaminant, is a significant health hazard. The kidney is one of the primary sites of injury after chronic Cd exposure. Kidney-based risk assessment establishes the urinary Cd threshold at 5.24 μg/g creatinine, and tolerable dietary intake of Cd at 62 μg/day per 70-kg person. However, cohort studies show that dietary Cd intake below a threshold limit and that tolerable levels may increase the risk of death from cancer, cardiovascular disease, and Alzheimer’s disease.

**Objective:**

We evaluated if the current tolerable dietary Cd intake guideline and urinary Cd threshold limit provide sufficient health protection.

**Discussion:**

Staple foods constitute 40–60% of total dietary Cd intake by average consumers. Diets high in shellfish, crustaceans, mollusks, spinach, and offal add to dietary Cd sources. Modeling studies predict the current tolerable dietary intake corresponding to urinary Cd of 0.70–1.85 μg/g creatinine in men and 0.95–3.07 μg/g creatinine in women. Urinary Cd levels of < 1 μg/g creatinine were associated with progressive kidney dysfunction and peripheral vascular disease. A urinary Cd of 0.37 μg/g creatinine was associated with breast cancer, whereas dietary Cd of 16–31.5 μg/day was associated with 25–94% increase in risk of estrogen receptor–positive breast cancer.

**Conclusion:**

Modeling shows that dietary intake levels for Cd exceed the levels associated with kidney damage and many other adverse outcomes. Thus, the threshold level of urinary Cd should be re-evaluated. A more restrictive dietary intake guideline would afford enhanced health protection from this pervasive toxic metal.

**Citation:**

Satarug S, Vesey DA, Gobe GC. 2017. Health risk assessment of dietary cadmium intake: do current guidelines indicate how much is safe? Environ Health Perspect 125:284–288; http://dx.doi.org/10.1289/EHP108

## Introduction

Cadmium (Cd) is a nonessential metal, a food-chain contaminant, and a constituent of cigarette smoke and polluted air (IPCS 1992). Diet is a major source of Cd exposure for nonsmokers, while cigarette smoke is an additional source for smokers (Satarug et al. 2013). Cd accumulates in the kidneys. Currently, human exposure to Cd is assumed, primarily, to damage the kidneys, especially the proximal tubular cells where the metal selectively concentrates. Consequently, kidney-based assessment of Cd exposure is often applied, with urinary Cd levels as indicators of Cd exposure. Other means of measuring Cd exposure relate to dietary intake estimates. Cancer risk assessment by the International Agency for Research on Cancer (IARC 1993) established Cd as a human lung carcinogen. Non-cancer risk assessment by the Food and Agriculture Organization/World Health Organization (FAO/WHO 1989, 1993, 2010) established a tolerable exposure and urinary threshold level that should protect against kidney damage. However, a wide diversity of Cd toxicity levels is increasingly apparent from recent studies, including the U.S. National Health and Nutrition Examination Survey (NHANES) (Hyder et al. 2013; Lin et al. 2013, 2014). Challenging kidney-based assessment is the observation that the carcinogenic effects of Cd appear to occur at exposure levels below the levels associated with kidney effects.

In this article, we highlight the basis of risk assessment for dietary Cd intakes together with dietary Cd intake estimates, derived from dietary and modeling studies. We provide evidence supporting the use of urinary Cd, a measure of cumulative lifetime exposure (body burden), in risk assessment, as opposed to the use of dietary intake estimates. We reviewed cross-sectional and longitudinal studies (published between 2010 and 2016) that link current dietary Cd intake levels to kidney damage, chronic kidney disease (CKD), cancer, and many other adverse health outcomes.

## Kidney Threshold Risk Assessment

In 1989, the Joint FAO/WHO Expert Committee on Food Additives (JECFA) established the safe dietary intake guideline, known as the Provisional Tolerable Weekly Intake (PTWI), defined as an estimate of the amount of the chemical with no intended function that can be ingested weekly over a lifetime without appreciable health risk (FAO/WHO 1989). The original PTWI for Cd was set at 400–500 μg per person per week, based on kidney critical concentration of Cd at 200 μg/g kidney wet weight, attainable after dietary Cd exposure of 140–260 μg/day for over 50 years or 2,000 mg of Cd over a lifetime (FAO/WHO 1989). It is considered that kidney damage by Cd causes a reduction in tubular re-absorptive function, evident from excess urinary excretion of nutrients (amino acids, glucose, zinc, calcium) and low molecular weight proteins, notably β2-microglobulin (β2-MG), retinol binding protein (RBP). Wallin et al. (2014) observed a positive correlation between kidney Cd levels and urinary α1-microglobulin (α1-MG) levels, and they suggested that urinary α1-MG could serve as a more sensitive biomarker for kidney toxicity, compared with other injury biomarkers like kidney injury molecule-1 (KIM-1), RBP, and β2-MG, which do not correlate with kidney Cd levels in chronic low-level exposure conditions. The original PTWI for Cd was later revised to 7 μg/kg body weight per week (70 μg/day for a 70-kg person) (FAO/WHO 1993). The current tolerable exposure was set at 25 μg/kg body weight per month (62 μg/day for a 70-kg person), and a urinary threshold of 5.24 μg/g creatinine (FAO/WHO 2010).

## Dietary Cd Intake Estimates for Swedish and French Populations

In the Swedish National Food Consumption Survey, Sand and Becker (2012) reported a dietary Cd intake of 10.6 μg/day for an average consumer. In this group, 40–50% of the Cd came from staple foods such as potatoes and wheat. For the high-Cd consumer, that is exposure above the 95th percentile, intake was 23 μg/day with additional Cd reportedly coming from seafood and spinach. Data derived from the second French Total Diet Study (TDS) (Arnich et al. 2012) showed the dietary Cd intake of 11.2 μg/day for an average consumer and 18.9 μg/day for the high consumer. For the average consumer, 35% came from bread products and another 26% from potato-based products. The additional Cd came from consumption of mollusks and crustaceans in the high-consumer group. Dietary Cd exposure in the second TDS (2007–2009) was four times higher than that of the first TDS (2000–2004), while the Cd concentrations were reported for the different food groups: Crustaceans and mollusks had the highest Cd content (0.167 mg/kg), followed by offal (0.053 mg/kg), sweet and savory biscuits (0.030 mg/kg) and cereal bars, and chocolate (0.029 mg/kg). However, data on variations in dietary habits were not reported. Likewise, the TDS did not report the body status of essential metals, which governs Cd absorption, or resultant toxicity. A study of 1,764 post-menopausal Danish women indicated leafy vegetables and soy-based products to be dietary Cd sources, but such dietary intake estimates (average 14 μg/day) marginally correlated with urinary Cd levels (Vacchi-Suzzi et al. 2015). These findings suggest such dietary intake estimates to be of limited use in health risk assessment.

## Urine Cd Concentration as a Measure of Cumulative Lifetime Cd Intake

Use of urinary Cd concentration, as a measure of cumulative lifetime exposure and body burden, has its foundation in the Cd-toxicokinetics model, developed by Kjellström and Nordberg (1978) from Swedish autopsy data. In a recent analysis of Cd levels in kidney, blood, and urine samples from 109 living kidney donors (mean age 51 years, mean kidney Cd 12.9 μg/g wet weight), Akerstrom et al. (2013) found a urine-to-kidney Cd ratio of 1:60, and urinary Cd of 0.42 μg/g creatinine corresponded to kidney Cd of 25 μg/g kidney. Assuming a urine-to-kidney Cd ratio of 1:20, urinary Cd of 1.25 μg/g creatinine corresponded to 25 μg/g Cd per kidney wet weight, comparable with Cd levels found in kidney cortex samples from Australians, 41–50 years of age (Satarug et al. 2002). Using the 1:20 ratio, the current WHO urinary Cd threshold of 5.24 μg/g creatinine (FAO/WHO 2010) corresponds to kidney Cd > 100 μg/g kidney weight, the levels seen mostly in workers exposed to high-Cd doses via inhalation. In the general population, blood Cd is considered a good estimate of body burden because population blood Cd levels correlate with urine Cd levels (Tellez-Plaza et al. 2010; Wu et al. 2014). Blood Cd is a better estimate of exposure for the elderly, people with diabetes, hypertension, and heavy smokers because the high prevalence of kidney dysfunction in these people may bias associations between their urine Cd levels and health outcomes.

## Model-Based Prediction of Urinary Cd Excretion at Tolerable Intake Guideline

Reverse dosimetry theory dictates that dietary Cd exposure and urinary Cd levels can be derived from a Cd toxicokinetic model, which describes mathematical relationships among the parameters, influencing Cd body burden, such as absorption rate, tissue distribution, half-life, and elimination rate. The original Cd-toxicokinetic model predicts that a Cd level of 50 μg/g kidney cortex wet weight corresponds to urinary Cd excretion of 2–4 μg/day, attainable after 50-year intake of dietary Cd at the tolerable weekly intake rate. A simulation model of Cd-toxicokinetics has been developed as a tool kit for prediction of Cd intake via oral (diet, water) versus inhalation (cigarette smoke, air) routes as a function of age and sex (Ruiz et al. 2010). Such simulation models predict that dietary intake of Cd at current tolerable monthly intake rate for 50 years will result in urinary Cd of 0.70–1.85 μg/g creatinine in men and 0.95–3.07 μg/g creatinine in women (Satarug et al. 2013). These urinary Cd levels, derived from modeled tolerable intake of dietary Cd, have been associated with kidney damage and CKD (Ferraro et al. 2010), concurrent with death from cancer (Lin et al. 2013; Adams et al. 2012), liver-related disease (Hyder et al. 2013), cardiovascular disease (CVD), ischemic heart disease, coronary heart disease (Tellez-Plaza et al. 2012b), and Alzheimer’s disease (Min and Min 2016). Tables 1 and 2 show details of population-based studies, giving evidence of Cd intake and exposure biomarkers associated with various adverse outcomes.

**Table 1:** Adverse outcomes associated with Cd exposure in cross sectional studies.

Outcomes/study	Risk estimates
Kidney damage^*a*^ and chronic kidney disease (CKD), Ferraro et al. (2010), NHANES 1999–2006, *n = *5,426, aged ≥ 20 years.	Blood (urinary) Cd levels > 1 μg/L were associated with kidney damage (OR 1.41, 95% CI: 1.10, 1.82), and CKD (OR 1.48, 95% CI: 1.01, 2.17).
Kidney damage and diminished kidney function^*b*^ (GFR), Lin et al. (2014), NHANES 2011–2012, *n = *1,545, aged ≥ 20 years.	Blood Cd levels > 0.53 μg/L were associated with kidney damage (OR 2.04, 95% CI: 1.13, 3.69), and diminished kidney function (OR 2.21, 95% CI: 1.09, 4.50).
Peripheral arterial disease (PAD), Tellez-Plaza et al. (2010), NHANES 1999–2004, *n = *6,456, aged ≥ 40 years, male mean urinary Cd levels of 0.31 μg/g creatinine (0.37 μg/L), and female mean urinary Cd levels of 0.44 μg/g creatinine (0.31μg/L).	Urinary Cd levels ≥ 0.69 μg/g creatinine were associated with male PAD (OR 4.90, 95% CI: 1.55, 15.54), and female PAD (OR 0.56, 95% CI: 0.18, 1.71). PAD risk in male nonsmokers increased with blood Cd levels, but PAD prevalence and blood Cd levels in female nonsmokers showed a U-shape, reflecting adverse effects at blood Cd levels < 0.3 μg/L.
Liver inflammation, NAFLD, and NASH, Hyder et al. (2013), NHANES III (1988–1994), *n = *12,732, aged ≥ 20 years.	Urinary Cd levels ≥ 0.83 μg/g creatinine were associated with female liver inflammation (OR 1.26, 95% CI: 1.01, 1.57). Urine Cd levels ≥ 0.65 μg/g creatinine were associated with male liver inflammation (OR 2.21, 95% CI: 1.64, 3.00), NAFLD (OR1.30, 95% CI: 1.01, 1.68) and NASH (OR 1.95, 95% CI: 1.11, 3.41).
Neurocognitive outcomes, Ciesielski et al. (2013), NHANES III (1988–1994), *n = *5,572, aged 20–59 years.	A 1 μg/L increase in urinary Cd was associated with a 1.93% (95% CI: –0.05, –3.81) reduction in a neurocognitive function test for attention/perception domain in nonsmokers.
Depression, Scinicariello and Buser (2015), NHANES 2007–2010, *n = *2,892, aged 20–39 years.	Blood Cd levels ≥ 0.54 μg/L were associated with depressive symptoms in nonsmokers (OR 2.91, 95% CI: 1.12, 7.58) and in smokers (OR 2.69, 95% CI: 1.13, 6.42).
Age-related macular degeneration (AMD), Wu et al. (2014), NHANES 2005–2008, *n = *5,390, aged ≥ 40 years.	Blood Cd levels ≥ 0.66 μg/L were associated with AMD (OR 1.56, 95% CI: 1.02, 2.40), compared with blood Cd 0.14–0.25 μg/L. Urine Cd levels ≥ 0.35 μg/L were associated with AMD in non-Hispanic whites (OR 3.31, 95% CI: 1.37, 8.01).
Pre-diabetes, Wallia et al. (2014), NHANES 2005–2010, *n = *2,398, aged ≥ 40 years, without albuminuria, diabetes or CKD.	Urinary Cd levels > 1.4 μg/g creatinine were associated with prediabetes in nonsmokers. Urine Cd levels > 0.7–0.9 μg/g creatinine were associated with pre-diabetes in nonsmokers and smokers.
Breast cancer, Gallagher et al. (2010), Long Island, New York, *n = *100 cases, *n = *98 controls, NHANES 1999–2008, *n = *92 cases, *n = *2,884 controls.	Long Island, urinary Cd levels ≥ 0.6 μg/g creatinine were associated with breast cancer (OR 2.69, 95% CI: 1.07, 6.78). NHANES, urinary Cd levels ≥ 0.37 μg/g creatinine were associated with breast cancer (OR 2.50, 95% CI: 1.11, 5.63).
Breast cancer, Itoh et al. (2014), Japan, *n = *309 cases, *n = *309 matched controls, mean age 53.8 years.	Dietary Cd intake levels ≥ 31.5 μg/day were associated with estrogen receptor positive (ER+) breast cancer (OR 1.94, 95% CI: 1.04, 3.63), compared with dietary Cd 21.4 μg/day.
Note: CI, confidence interval; HR, hazard ratio; NAFLD, non-alcoholic fatty liver; NASH, non-alcoholic steatohepatitis; *n*, sample size; OR, odds ratio. ^***a***^Urinary albumin to creatinine ratio ≥ 30 mg/g creatinine. ^***b***^Estimated glomerular filtration rate (eGFR) < 60 mL/min/1.73 m^2^.	

**Table 2:** Adverse outcomes associated with Cd exposure in longitudinal studies.

Outcomes/study	Risk estimates
Breast cancer, Julin et al. (2012), Swedish postmenopausal women, 12.2-year follow-up, *n = *55,987.	Dietary Cd levels ≥ 16 μg/day were associated with breast cancer (RR 1.27, 95% CI: 1.07, 1.50), and estrogen receptor positive (ER+) breast cancer (RR 1.25, 95% CI: 1.03, 1.52).
Mortality from heart and vascular diseases, Tellez-Plaza et al. (2012b), NHANES 1999–2004, average 4.8-year follow-up, *n = *8,989.	Urinary Cd levels ≥ 0.57 μg/g creatinine were associated with death from CVD (HR 1.74, 95% CI: 1.07, 2.83), ischemic heart disease (HR 2.53, 95% CI: 1.54, 4.16), and coronary heart disease (HR 2.09, 95% CI: 1.06, 4.13), compared with urinary Cd levels ≤ 0.14 μg/g creatinine. Lowering Cd exposure by 4 fold could have prevented 8.8% of total deaths and 9.2% of CVD deaths.
Cancer mortality, Adams et al. (2012), NHANES III (1988–1994), average 13.4-year follow-up, *n = *7,455 men, *n = *8,218 women.	Urinary Cd levels ≥ 0.58 μg/g creatinine were associated with death from lung cancer in men (HR 3.22, 95% CI: 1.26, 8.25). A 2-fold rise in urinary Cd was associated with death from cancer in both men and women (male HR 1.26, 95% CI: 1.07, 1.48; female HR 1.21, 95% CI: 1.04, 1.42).
Mortality from liver-related diseases, Hyder et al. (2013), NHANES III (1988–1994), median 14.6-year follow-up, *n = *12,732.	Female urinary Cd levels ≥ 0.83 μg/g creatinine and male urine Cd levels ≥ 0.65 μg/g creatinine were associated with death from liver-related diseases (HR 3.42, 95% CI: 1.12, 10.47).
Cancer mortality, Lin et al. (2013), NHANES III (1988–1994), 12.4-year follow-up, *n = *5,204.	Urinary Cd levels > 0.79 μg/g creatinine were associated with cancer death in men (HR 3.13, 95% CI: 1.88, 5.20). Urinary Cd levels > 1.05 μg/g creatinine were associated with cancer death in women (HR 1.65, 95% CI: 1.13, 2.41).
All-cause mortality, Patel et al. (2013), NHANES 1999–2004, median 2.5–5.8-year follow-up, *n = *22,076.	A 1-SD change in logged^*a*^ urinary Cd levels was associated with mortality (HR 1.6, 95% CI: 1.3, 2.0). A 1-SD change in logged blood Cd levels was associated with mortality (HR 1.4, 95% CI: 1.2, 1.6). Three other factors associated with death were low-level physical activity, smoking, and low serum lycopene (a dietary antioxidant).
Mortality from Alzheimer’s disease, Min and Min (2016), NHANES (1999–2004), followed up until 31 December 2011, *n = *4,060, aged ≥ 60 years.	Blood Cd levels > 0.6 μg/L were associated with death from Alzheimer’s disease (HR 3.83, 95% CI: 1.39, 10.59), compared with blood Cd levels < 0.3 μg/L. Higher-blood Cd levels at baseline were associated with a marginal increase in death from all causes (*p* = 0.07).
Note: CVD, cardiovascular disease; CI, confidence interval; HR, hazard ratio; RR, rate ratio; SD, standard deviation. ^***a***^Logarithmic transformation.	

## Cross Sectional Studies

Table 1 is a summary of cross-sectional and case-control studies of Cd exposure outcomes. In NHANES 1999–2006 (Ferraro et al. 2010), blood and urinary Cd levels > 1 μg/L were associated with kidney damage [OR 1.41, 95% confidence interval (CI): 1.10, 1.82] and CKD (OR 1.48, 95% CI: 1.01, 2.17). An association of Cd exposure and CKD became obscured when creatinine was used to correct for diluting effects of spot urine samples (Ferraro et al. 2010). This may indicate variability in creatinine secretion by kidney. In NHANES 2011–2012 (Lin et al. 2014), blood Cd levels > 0.53 μg/L were associated with kidney damage (OR 2.04, 95% CI: 1.13, 3.69) and low GFR (OR 2.21, 95% CI: 1.09, 4.50), and risk of Cd-induced kidney damage was particularly high (OR 3.38, 95% CI: 1.39, 8.28) in the participants who had lower zinc status, compared with those with higher zinc status. In NHANES 1999–2004 (Tellez-Plaza et al. 2010), urinary Cd levels ≥ 0.69 μg/g creatinine were associated with peripheral arterial disease (PAD) in men (OR 4.90, 95% CI: 1.55, 15.54), and in women (OR 0.56, 95% CI: 0.18, 1.71). Further, PAD risk in male nonsmokers increased with blood Cd levels, but PAD prevalence and blood Cd levels in female nonsmokers showed a U-shape relation, reflecting effects at blood Cd levels below 0.3 μg/L. In NHANES III (1988–1994), Hyder et al. (2013) found that urinary Cd levels ≥ 0.83 μg/g creatinine were associated with liver inflammation in women (OR 1.26, 95% CI: 1.01, 1.57), while urinary Cd levels ≥ 0.65 μg/g creatinine were associated with liver inflammation (OR 2.21, 95% CI: 1.64, 3.00), non-alcoholic fatty liver (OR 1.30, 95% CI: 1.01, 1.68) and non-alcoholic steatohepatitis (OR 1.95, 95% CI: 1.11, 3.41) in men. Ciesielski et al. (2013) found a 1 μg/L increment in urinary Cd was associated with a 1.93% reduction in a neurocognitive test for attention/perception domain among nonsmokers in NHANES III. In NHANES 2007–2010 (Scinicariello and Buser 2015), blood Cd levels ≥ 0.54 μg/L were associated with depressive symptoms in nonsmokers (OR 2.91, 95% CI: 1.12, 7.58), and smokers (OR 2.69, 95% CI: 1.13, 6.42). In NHANES 2005–2008, Wu et al. (2014) found blood Cd levels ≥ 0.66 μg/L were associated with age-related macular degeneration (AMD) (OR 1.56, 95% CI: 1.02, 2.40). The Cd and AMD association was particularly strong in non-Hispanic whites with urinary Cd levels ≥ 0.35 μg/L (OR 3.31, 95% CI: 1.37, 8.01). In NHANES 2005–2008 (Wallia et al. 2014), urinary Cd levels > 1.4 μg/g creatinine were associated with risk of prediabetes among nonsmokers. In NHANES 1999–2008, Gallagher et al. (2010) found urinary Cd levels ≥ 0.37 μg/g creatinine were associated with breast cancer among women (OR 2.50, 95% CI: 1.11, 5.63). In another study, Itoh et al. (2014) found dietary Cd intake levels ≥ 31.5 μg/day were associated with estrogen receptor positive (ER+) breast cancer in Japanese women (OR 1.94, 95% CI: 1.04, 3.63).

## Longitudinal Studies

Table 2 is a summary of longitudinal studies of Cd exposure outcomes. In a Swedish cohort (Julin et al. 2012), dietary Cd intake levels ≥ 16 μg/day were associated with breast cancer (RR 1.27, 95% CI: 1.07, 1.50), and ER+ breast cancer (RR 1.25, 95% CI: 1.03, 1.52). In NHANES 1999–2004 follow-up (Tellez-Plaza et al. 2012b), urinary Cd levels ≥ 0.57 μg/g creatinine were associated with death from CVD (HR 1.74, 95% CI: 1.07, 2.83), ischemic heart disease (HR 2.53 95% CI: 1.54, 4.16), and coronary heart disease (HR 2.09, 95% CI: 1.06, 4.13). Population attributed risks suggest that reduction in urinary Cd from 0.57 to 0.14 μg/g creatinine could prevent 8.8% overall deaths and 9.2% CVD deaths. An equivalent analysis using blood Cd data gives parallel results; a reduction of blood Cd from 0.80 to 0.22 μg/L could prevent 7% overall deaths and 7.5% CVD deaths. In NHANES III follow-up, Adams et al. (2012) found urinary Cd levels ≥ 0.58 μg/g creatinine were associated with death from lung cancer in men (HR 3.22, 95% CI: 1.26, 8.25), while Hyder et al. (2013) found female urinary Cd levels ≥ 0.83 μg/g creatinine, and male urinary Cd levels ≥ 0.65 μg/g creatinine were associated with death from liver-related diseases (HR 3.42, 95% CI: 1.12, 10.47). Also in NHANES III follow-up (Lin et al. 2013), urinary Cd levels > 0.79 μg/g creatinine were associated with cancer death in men (HR 3.13, 95% CI: 1.88, 5.20), while urinary Cd levels > 1.05 μg/g creatinine were associated with cancer death in women (HR 1.65, 95% CI: 1.13, 2.41). In the NHANES 1999–2004 follow-up (Patel et al. 2013), a 1-SD change in logged exposure levels was associated with death from all causes (HR 1.6, 95% CI: 1.3, 2.0 for urinary Cd, and HR 1.4, 95% CI: 1.2, 1.6 for blood Cd). In the NHANES 1999–2004 follow-up (Min and Min 2016), blood Cd levels > 0.6 μg/L were associated with death from Alzheimer’s disease (HR 3.83, 95% CI: 1.39, 10.59).

## Discussion

Chronic intake of low-level dietary Cd has long been viewed as a subtle, long term and non-specific impairment. In contrast, such low-level dietary Cd intake has now been implicated in more serious health outcomes than previously perceived. Of concern, NHANES data indicate a significant proportion of the U.S. population is at risk of adverse effects from low-level dietary Cd intakes. Data from the NHANES 1999–2008 participants, aged 20–85 years, indicate Cd exposure prevalence of 94–98% in nonsmokers, and 96–99% in smokers (Riederer et al. 2013). A decline in Cd exposure in the U.S. over the 20-year (1988–2008) period could only be attributed to a reduction in smoking prevalence with little evidence to suggest a reduction in dietary Cd sources (Tellez-Plaza et al. 2012a). Overall Cd exposure prevalence among NHANES 2007–2012 participants, aged ≥ 20 years remains as high as 91.9% (Buser et al. 2016). These high exposure prevalence rates suggest that even a small increase in disease risk by Cd exposure can result in a large number of people affected by a disease that is preventable. In the NHANES 1999–2006, overall (female) prevalence of urinary Cd > 1, > 0.7 and > 0.5 μg/g creatinine among ≥ 20-year nonsmokers without CKD was 1.7 (2.5)%, 4.8 (7.1)%, and 10.8 (16)%, respectively (Mortensen et al. 2011). These data are a cause for concern because urinary Cd levels ≥ 0.37 to ≥ 0.65 μg/g creatinine have been associated with female breast cancer (Gallagher et al. 2010), death from heart disease (Tellez-Plaza et al. 2012b), death from cancer (Adams et al. 2012; Lin et al. 2013), and liver-related diseases (Hyder et al. 2013). Further, the prevalence of diminished kidney function among the NHANES 2011–2012 participants of 7.4% exceeds the 5% acceptable disease prevalence (Lin et al. 2014). Thus, restrictive dietary intake guidelines are required to safeguard against a further increase in dietary Cd intake.

## Conclusion

Current population risk assessment of dietary Cd intake relies on estimates of dietary Cd intake and/or maintenance of threshold levels of urinary Cd that should protect the kidney from Cd-induced damage. Risk assessment using dietary Cd intake estimates has been questioned because they show only a marginal correlation with urinary Cd levels, a well-founded measure of lifetime intakes. Blood Cd levels, however, show a correlation with urinary Cd levels, and they could thus be of value in risk assessment; blood Cd levels ≥ 1 μg/L were associated with CKD, while blood Cd levels above 0.5 μg/L were associated with AMD, depression, and death from Alzheimer’s disease. Using a Cd-toxicokinetic simulation model, we have found that current tolerable dietary intake guidelines do not contain a safety margin, given that the modeled dietary intake levels exceed the levels associated with kidney damage and many other adverse health outcomes seen in cohorts and cross-sectional studies. These data point to the need for a revision of tolerable dietary intake levels for Cd, and public measures to minimize the food-chain contamination by Cd. Risk reduction measures, supported by international food legislation, should not be relaxed. A maximally permissible concentration (MPC) for Cd in foods should be set as low as reasonably achievable. Current MPC for rice is set at 0.4 mg/kg dry grain weight, but global risk assessment suggests 0.1 mg/kg is necessary. Persistence of Cd in the environment, coupled with its high soil-to-plant transfer rates, requires long-term management of Cd in the environment (soil, air, and water), and in agriculture, where consideration should be given to leafy salad vegetables, such as spinach and lettuce, which are known to be hyper accumulators of Cd. In the absence of non-toxic chelating agents to reduce Cd tissue burden, maintenance of the lowest Cd levels in food crops is pivotal.
